# Occupation and risk of sudden death in a United States community: a case–control analysis

**DOI:** 10.1136/bmjopen-2015-009413

**Published:** 2015-12-18

**Authors:** Lin Zhang, Kumar Narayanan, Vallabh Suryadevara, Carmen Teodorescu, Kyndaron Reinier, Audrey Uy-Evanado, Harpriya Chugh, Zhi-Jie Zheng, Karen Gunson, Jonathan Jui, Sumeet S Chugh

**Affiliations:** 1 Shanghai Jiaotong University School of Public Health, Shanghai, China; 2 The Heart Institute, Cedars-Sinai Medical Center, Los Angeles, California, USA; 3 Department of Pathology, Oregon Health and Science University, Portland, Oregon, USA; 4 Department of Emergency Medicine, Oregon Health and Science University, Portland, Oregon, USA

**Keywords:** CARDIOLOGY, EPIDEMIOLOGY, occupation, sudden cardiac death

## Abstract

**Objective:**

Work environment is said to influence cardiovascular risk. We assessed whether nature of occupation affects risk of sudden cardiac death (SCD) in the general population.

**Methods:**

In the ongoing, prospective Oregon Sudden Unexpected Death Study (catchment population 1 million), working-age SCD cases (18–65 years) were compared with controls who died from any cause. Usual occupation obtained from death certificates was classified using the US Census Bureau standard occupational classification descriptions and categorised as white collar, blue collar or homemaker. Odds ratio (OR) for SCD by occupation category was obtained and clinical profile of SCD cases was compared by occupation type.

**Results:**

Among SCD cases (n=646; 74% male) compared to controls (n=622; 73.6% male), the proportion of white collar workers was higher among male SCD cases (52.7% vs 43.7%; p=0.01); the difference in females was smaller (59.5% vs 55%; p=0.62). Adjusting for race and smoking status, male white collar workers had a higher risk of SCD compared to blue collar workers (OR=1.67, (1.26 to 2.23), p<0.001). A similar, non-significant trend was observed among females (OR 1.49 (0.81 to 2.75); p=0.20). White collar SCD cases were *less* likely to be current smokers (34.7% vs 45.3%, p=0.008), drug misusers (13.1% vs 18.5%) or have diabetes (21.4% vs 28.2%, both p=0.07) compared to blue collar workers. Other cardiac risk factors were similar.

**Conclusions:**

A white collar occupation was associated with increased risk of SCD, when compared to blue collar occupations. Since differences in conventional risk factors did not explain this elevated risk, work-related behavioural and psychosocial stressors warrant a closer evaluation.

**Strengths and limitations of this study**

- To the best of our knowledge, this is the first United States study to examine the specific association between occupational status and the risk of sudden cardiac death (SCD) in the general population.
- Individual-level data have been analysed for working-age out-of-hospital SCD cases compared to frequency matched (age, gender, year of death and geographical location) overall mortality controls across 7 years of ascertainment.
- Since the analysis was conducted among deceased individuals (at least 90% of all patients presenting with sudden cardiac arrest will not survive), questionnaires or in-person interviews could not be conducted.

## Introduction

In the United States, at least 300 000 cases of sudden cardiac death (SCD) occur every year, accounting for 50% of cardiovascular mortality.^1^ Despite an overall decline in cardiovascular morbidity and mortality over the past several decades, survival from resuscitated sudden cardiac arrest remains in the range of 5%.^2^ Given the dramatically low rate of survival, it is crucial to enhance prediction and prevention of this devastating problem by addressing fundamental aspects of epidemiology.

The important association of the work environment with health and disease is well recognised. Occupational status can determine psychosocial factors such as job-related activity, responsibility and stress,^3–5^ and also predicts socioeconomic status.^6^ Occupation status may predict health outcomes, including all-cause mortality,^3^ ^7^ prevalence of risk factors for cardiovascular disease^8–10^ and incidence of cardiovascular disease.^4^ ^11^ Furthermore, a potential link between work-related stress and adverse cardiovascular outcomes has been suggested; for instance, an association between law enforcement/firefighting occupations and sudden death while on duty has been reported.^12^ ^13^ Overall, cardiovascular morbidity is also higher among on-duty police officers than the general population.^14^ However, there is a significant lack of studies on whether occupational status influences risk of SCD in the general population. Understanding how different occupations affect SCD risk could be important from a public health perspective, enabling the design of targeted preventive interventions at the community level.

In the present study, we compared the occupation status of SCD cases identified from the general population to control subjects who died from any cause (overall mortality) in the same area and during the same time period. Second, we evaluated the relationship between occupation status and different cardiovascular risk factors among patients with SCD in order to better understand how occupation type may be linked to SCD risk.

## Methods

### Study populations

This analysis was conducted as part of the Oregon Sudden Unexpected Death Study (Oregon SUDS) between 1 January 2006 and 31 December 2013. The Oregon SUDS is an ongoing, prospective, population-based study of out-of-hospital SCD cases in the Portland, Oregon metropolitan area (catchment population approximately 1 million). Detailed descriptions of subject recruitment and methodology have been published previously.^15^ ^16^ In brief, possible SCD cases were prospectively ascertained through liaison with the first responders (fire department, local ambulance and local hospital emergency rooms) and the county medical examiner. After a detailed review of available medical records, autopsy reports and the circumstances of arrest, SCD cases were identified through a three-physician adjudication system. SCD was defined as an unexpected death without obvious extracardiac cause, occurring with a rapidly witnessed collapse, or if unwitnessed, occurring within 24 h of the patient being seen in their usual state of health.^17^ Patients with known terminal illnesses (such as cancer), non-cardiac causes of sudden death (such as cerebrovascular accident) and drug overdose were excluded. For the purpose of analysing the association between SCD and occupation class, cases were required to be between 18 and 65 years of age with death certificate information available.

During the same time period, a control group of subjects who died from any cause (overall mortality) was ascertained from the Oregon Vital Statistics System mortality database. Overall mortality was defined as the total number of deaths due to any cause during the given time period (2006–2013). Patients who died from any cause were chosen as controls so as to be able to specifically assess the association between occupation and SCD rather than a non-specific association with overall mortality. Controls were ascertained in a random manner, frequency-matched by age, gender, year of death and county of residence in a 1:1 ratio with cases. Manner of death for controls, as recorded on the death certificate, was: 75% natural, 23% accidental/suicide, 0.5% homicide and 1% undetermined. The most common causes of death were malignant neoplasms (n=147, 24%) and diseases of the circulatory system (n=117, 19%), based on International Classification of diseases 10th Edition (ICD-10) mortality codes on the death certificate. The circulatory system causes included atherosclerotic heart disease or chronic ischaemic heart disease (n=44, 6.8%), acute myocardial infarction (n=19, 3.1%), cardiac arrest/cardiac arrhythmia/ventricular fibrillation (n=5, <1%), valvular heart disease (n=5, <1%), cardiomyopathies (n=5, <1%) and heart failure (n=6, 1%). Information on age, sex, race, occupation, industry, years of education, marital status and tobacco use was obtained from the death certificate.

## Measures

### Assessment of occupation status

Occupation status was classified by a comprehensive review of the individuals’ occupation and industry/business based on death certificate records. The occupation recorded on the death certificate is the individual's usual work performed during most of his/her working life. Each occupation was classified into one of three prespecified categories, namely *white collar jobs*, *blue collar jobs*, or *homemaker* based on an objective assessment of job characteristics. Classification was guided by the US Census Bureau standard occupational classification descriptions.^18^ White collar jobs included patients who mainly performed non-manual work including professional, administrative, managerial and higher technical workers, as well as office and sales/service workers. Blue collar jobs included patients who mainly performed manual labour including: construction, production, maintenance, transportation and other labour workers. Individuals who were recorded as self-employed were considered either white collar or blue collar based on the nature of the job and industry. Business owners and operators, as well as contractors, were included in white collar jobs as were those doing managerial and administrative work; manufacturers and handymen were included in blue collar jobs. Occupations recorded as student, disabled and unclassified, and those missing occupation information were excluded from this study. The occupation assessment was performed independently by two reviewers blinded to the case–control status of patients. Disagreements in classification were resolved by discussion and finally arbitrated by the corresponding author.

### Assessment of individual demographics

On the basis of death certificate records, education was classified into four categories: less than high school (<12th grade), high school degree (=12th grade with high school diploma), some college credit (13–15th grades) and bachelor degree or higher (≥16th grade). Smoking, alcohol consumption and recreational drug use were defined as either current use or no current use (never or former use).

### Conventional risk factors for SCD cases

For SCD cases, clinical data are collected from available medical records by a dedicated team of physician researchers. Access to these data is made possible by collaboration with the area emergency medical services, medical examiner's (coroner's) office as well as local hospitals and the Kaiser health maintenance organization (HMO). Clinical information on conventional risk factors was obtained from available medical records prior and unrelated to the SCD event for SCD cases, as recorded in the Oregon SUDS database. The following comorbidities were included: coronary artery disease (CAD), diabetes, hypertension, hyperlipidaemia, obesity, chronic renal insufficiency and chronic obstructive pulmonary disease. CAD was defined as ≥50% stenosis in a major coronary artery, history of myocardial infarction or history of coronary revascularisation. Obesity was defined as a body mass index (body mass index=weight/height^2^) of ≥30 kg/m^2^.^19^ The requirement for informed consent was waived as the study only involved analysis of de-identified data in deceased individuals.

### Statistical analysis

Continuous and categorical variables were expressed as mean±SD and number (percentage), respectively. Independent samples t test and χ^2^ test or Fisher’s exact test were used for comparison of continuous and categorical variables. Multivariable logistic regression analyses were used to estimate the ORs for SCD associated with occupation status. Analyses were adjusted for potential confounders which were significantly different in bivariate case–control comparisons. A two-tailed p value of ≤0.05 was considered statistically significant. Statistical analysis was performed using SPSS V.20.0 (IBM Corporation, New York, USA).

All authors had full access to all of the data (including statistical reports and tables) in the study and can take responsibility for the integrity of the data and the accuracy of the data analysis.

## Results

### Clinical and demographic characteristics

**Figure1:**
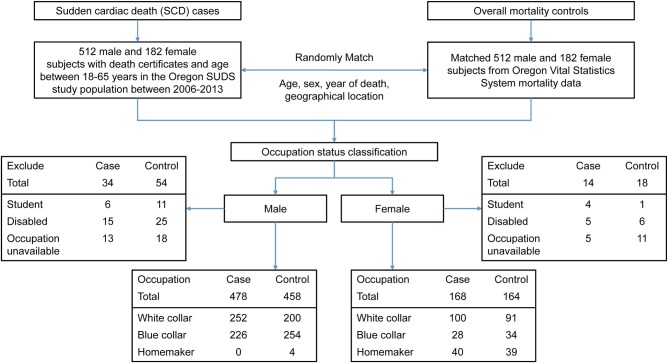
Flow chart of the study population.

Between January 2006 and December 2013, 512 male and 182 female cases of SCD, 18–65 years of age, were identified in the Portland, OR metropolitan area (figure 1). Of these, 478 male cases (93.3%) and 168 female cases (92.3%) were included in this study. Reasons for exclusion were employment status recorded as students (6 male, 4 female), disabled (15 male, 5 female) or unclassified/missing occupations (13 male, 5 female). In the same time period, 512 male controls and 182 female controls were frequency-matched by age, sex and year of death from the overall mortality population, with a total of 458 male controls (89.4%) and 164 female controls (90.2%) included for analysis. Controls were excluded if recorded as students (11 male, 1 female), disabled (25 male, 6 female) or unclassified/missing job title (18 male, 11 female).

Clinical/demographic characteristics of the cases and controls are shown in table 1. Among both males and females, SCD cases had a significantly higher proportion of current smokers (male 41% vs 15.3%, p<0.001; female 29.8% vs 12.8%, p=0.001) than overall mortality controls. Moreover, female SCD cases were more likely to be African-American (8.3% vs 2.4%, p=0.02). Among both males and females, there were no significant case–control differences in age (male 50.2±9.8 vs 49.5±10.2 years; p=0.24; female 51.1±11.0 vs 51.2±10.1 years; p=0.95) and educational attainment (male p=0.59; female p=0.38).

**Table 1:** Distribution of occupation status and demographic characteristics among SCD cases and overall mortality controls

	Male			Female		
	Case (%)	Control (%)	p Value	Case (%)	Control (%)	p Value
Total numbers	478	458		168	164	
Age (years)	50.2±9.8	49.5±10.2	0.24	51.1±11.0	51.2±10.1	0.95
African-American	35 (7.3)	23 (5.0)	0.14	14 (8.3)	4 (2.4)	0.02
Current smoking	196 (41.0)	70 (15.3)	<0.001	50 (29.8)	21 (12.8)	0.001
Educational attainment			0.59			0.38
Less than high school	68 (14.2)	67 (14.6)		16 (9.5)	12 (7.3)	
High school	187 (39.1)	189 (41.3)		66 (39.3)	59 (36.0)	
Some college	127 (26.6)	126 (27.5)		54 (32.1)	49 (29.9)	
Bachelor or higher	96 (20.1)	76 (16.6)		32 (19.0)	44 (26.8)	
Occupation status			0.01			0.62
White collar	252 (52.7)	200 (43.7)		100 (59.5)	91 (55.5)	
Blue collar	226 (47.3)	254 (55.5)		28 (16.7)	34 (20.7)	
Homemaker	0	4 (0.8)		40 (23.8)	39 (23.8)	

Results presented as mean±SD for continuous variables and n (%) for categorical variables.

SCD, sudden cardiac death.

### Occupation status

**Figure 2:**
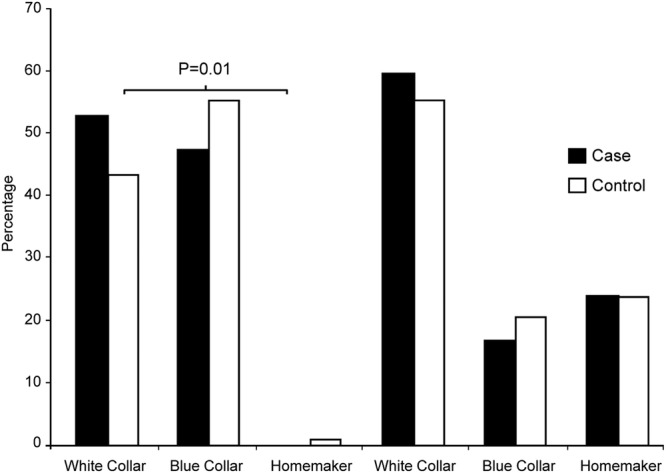
Comparison of the occupation status in cases and controls.

We observed significant differences in occupation status between SCD cases versus controls (figure 2). In males, there were significantly more white collar workers among SCD cases (52.7% vs 43.7%; p=0.01), with a very low frequency of homemakers overall (0 vs 0.8%). In females, similar, albeit smaller, differences in occupation status between cases and controls were observed, which did not reach statistical significance. More than half of the females were white collar workers (59.5% cases vs 55.5% controls; p=0.62), followed by homemakers (23.8% vs 23.8%) and blue collar workers (16.7% vs 20.7%; figure 2).

### Adjusted OR for SCD by occupation status

In the multivariable logistic regression analysis, compared with blue collar workers, there was a 1.67-fold increased risk of SCD events (95% CI (1.26 to 2.23); p<0.001) in male white collar workers, after adjusting for smoking status and African-American race. Similar trends were identified in females, but did not reach statistical significance (table 2).

**Table 2:** Adjusted ORs for SCD

	Male		Female	
	OR (95% CI)	p Value	OR (95% CI)	p Value
White collar workers (vs blue collar)*	1.67 (1.26 to 2.23)	<0.001	1.49 (0.81 to 2.75)	0.20
Current smoker	3.90 (2.79 to 5.44)	<0.001	3.10 (1.60 to 6.03)	0.001
African-American	1.39 (0.76 to 2.54)	0.28	3.34 (1.05 to 10.7)	0.04

*Model adjusted for African-American race and current smoker.

SCD, sudden cardiac death.

### Comparison of comorbidities among white collar and blue collar SCD cases

The clinical profiles and adverse health behaviours among SCD cases that were white collar or blue collar workers are shown in table 3. The prevalence of hypertension, hyperlipidaemia, obesity, CAD, chronic renal insufficiency and obstructive pulmonary disease was not significantly different between white collar and blue collar workers. However, white collar workers were less likely to be current smokers (34.7% vs 45.3%, p=0.008), current drug misusers (13.1% vs 18.5%; p=0.07) or to have diabetes (21.4% vs 28.2%, p=0.07), the latter two with borderline significance.

**Table 3:** Clinical profiles and adverse health behaviours in white collar versus blue collar workers among SCD cases

	White collar workers (N=352)	Blue collar workers (N=254)	p Value
Age (years)	50.8±8.9	50.2±9.7	0.42
Black race	29 (8.2%)	20 (7.9%)	0.71
Current smoker	122 (34.7%)	115 (45.3%)	0.008
Current alcohol use	39 (11.1%)	35 (13.8%)	0.31
Current drug misuser	46 (13.1%)	47 (18.5%)	0.07
Diabetes*	69 (21.4%)	66 (28.2%)	0.07
Hypertension*	140 (43.5%)	108 (46.2%)	0.53
Hyperlipidaemia*	88 (27.3%)	69 (29.5%)	0.58
Obesity*	66 (20.5%)	48 (20.5%)	0.99
Coronary artery disease†	157 (75.1%)	128 (81.5%)	0.14
Chronic renal insufficiency*	37 (11.5%)	32 (13.7%)	0.44
Obstructive pulmonary disease*	35 (10.9%)	36 (15.4%)	0.11

Results presented as mean±SD for continuous variables and n (%) for categorical variables.

*Data on diabetes, hypertension, hyperlipidaemia, obesity, chronic renal insufficiency and obstructive pulmonary disease were available for 322 white collar workers and 234 blue collar workers.

†Data on CAD were available for 209 white collar workers and 157 blue collar workers.

CAD, coronary artery disease; SCD, sudden cardiac death.

## Discussion

To the best of our knowledge, this is the first study to investigate the specific association between occupational status and the risk of SCD in the general population. In this community-based sample of SCD individuals, we evaluated the association of occupation status with the risk of SCD, by comparisons with individuals who died from any cause in the same geographic area and over the same time period. This design enabled us to evaluate whether certain occupational profiles are associated with SCD specifically, rather than a non-specific association with overall mortality. Although we cannot estimate absolute risk of SCD by occupation, we found that among males, there was a higher proportion of white collar workers in the SCD group compared with the control subjects. After adjusting for potential confounders, male white collar workers had a 1.67-fold increased risk of SCD compared with blue collar workers. A similar trend was seen in females, though it was not statistically significant, which may be related to the smaller number of female patients, consistent with the established higher risk of SCD in men.^16^ The relation of occupation as a potential risk factor for SCD would be of significant public health impact in the long term, given the overall population burden of SCD, which is higher than all cancers combined.^20^ Furthermore, we compared the clinical profiles and health behaviours among white collar and blue collar SCD cases. Interestingly, we found a slightly more favourable profile among white collar workers with a lower prevalence of current cigarette smoking and borderline lower prevalence of current drug abuse and diabetes mellitus. This suggests that the excess risk of SCD observed among white collar workers in this study may not be explained by differences in conventional risk factors. Our data did not allow an evaluation of physical activity level in white collar and blue collar workers, but conventional risk factors were similar between the two groups. Thus, while this is currently speculative, we raise the possibility that psychosocial stress-related factors may have played a role in the higher burden of SCD among white collar workers.

A few previous studies have suggested that blue collar workers may have higher cardiovascular morbidity/prevalence of CAD, findings that are potentially contrary to our findings.^21–25^ This contrast could be due to several factors that are worth discussing. The first relates to the setting of this study, both from the point of view of time period and geographic location. This relatively contemporary study was conducted in the developed world, in a region of the USA that may have other defining features. The Portland, Oregon metropolitan area has highly favourable demographic, education and health metrics even when compared to the US national average. The population is younger (median age 36 years) with a lower prevalence of obesity and smoking, and a higher prevalence of health-promoting behaviours.^26^ These factors are likely to have influenced our results by reflecting in relatively few differences of conventional cardiovascular risk factors between white collar and blue collar workers, bringing out a relatively greater contribution of psychosocial/stress-related variables. Clearly, these possibilities need further focused evaluation in additional studies. Our study may also be unique in the involvement of an entire population of approximately one million, enabling access to large numbers of SCD cases that could be compared to all-cause mortality in the same population, potentially identifying risk factors more specific for SCD. Thus, it could be postulated that occupation-related psychosocial stressors, arguably greater in executive professions, may trigger SCD events in susceptible individuals. Psychological stress in the workplace may exert an influence on CHD risk by acting as a ‘double hit’ in those at high risk of CAD.^27^ High job stress is defined as having a demanding job that provides limited opportunity for decision-making or use of one's creative or individual skills.^28^ Individuals with greater demands at work and lower decision latitude are prone to higher job stress which has been shown to contribute to increased risk for CHD.^29–35^ High job demand has been shown to be more frequently present in white collar workers.^27^ ^29^ However, some other studies did not find a significant association between individual job stress and incidence of cardiac death or non-fatal myocardial infarction.^36–40^

To extend this discussion further, there are several additional factors that could contribute to higher risk in white collar versus blue collar workers. In general, white collar workers work greater hours and experience greater mental stress compared with blue collar workers.^41^ Moreover, other emotionally stressful events such as anger could acutely trigger coronary events in vulnerable people with CHD.^42^ In addition, physical activity exhibits a close correlation with health indicators. White collar workers are in general more likely to have lower occupational physical activity than blue collar workers.^43^ White collar workers also have longer occupational sedentary time and more often work overtime than blue collar workers.^44^ Such sedentary behaviour can add to health risks including an increased risk of all-cause and cardiovascular mortality.^45^ A similar trend for association between occupation status and SCD was seen in women which was not statistically significant. This is most likely due to the relatively fewer numbers of women in our study and this association needs to be further evaluated in larger studies. The effect of occupation on cardiovascular and, in particular, SCD risk could have important implications for public health interventions that target the workplace. The American Heart Association has emphasised worksite-based cardiovascular disease prevention and the need for effective interventions to improve cardiovascular health among the working population.^46^ ^47^ Identification of potentially ‘high-risk profile’ occupation types may allow greater focus on such groups with more health education and promotion of cardioprotective behaviours to counter work-related stress. Incentive-based employee health programmes rewarding achievement of specific health goals in a timely fashion could serve as a means of using the workplace as a captive environment to improve cardiovascular health.^48^ ^49^

### Limitations

There are some potential limitations of this study. We selected individuals between 18 and 65 years of age and the results may not be generalisable to the entire population. However, as the common working age group, we felt that this was the most relevant subset to study. Observational studies may be influenced by confounders, though we attempted to choose a well-matched population and adjusted for other risk factors in multivariable models. The control group included all causes of mortality, providing a robust comparison group. While a small subgroup of controls died of sudden cardiac death, inclusion of individuals with sudden cardiac death in the control group would most likely lead to an underestimation of any associations reported, thereby strengthening our findings. In our study, we extracted occupation from death certificates. Occupation classification was based on the US Census Bureau descriptions and during the process, evaluators were blinded to case–control status to reduce the potential for systematic bias. Still, there exists some potential for misclassification of occupational status. However, we looked at risk in the broad context of white collar versus blue collar occupations which are likely to be applicable to similar jobs across the world. Additional studies in diverse populations are needed to evaluate the generalisability of our findings.

Moreover, we did not have detailed socioeconomic status information available which could have influenced the effect of occupation on SCD, though educational attainment was similar in cases and controls. In addition, studies have shown that the correlation between occupation and income may not be consistent.^6^

Finally, owing to the low survival rate of SCD in the general population, it was not possible to conduct personalised interviews to quantify job stress at the individual level. Further studies in diverse geographic and socioeconomic populations are needed to assess the generalisability of these findings. However, our study identifies a potential link between occupation-related stressors and SCD in the population, and if confirmed, these findings could pave the way for workplace-based interventions to modify cardiac risk.

## Conclusion

In this large US community, having a white collar job was specifically associated with increased risk of sudden death, in contrast to death from any cause. Differences in clinical profile did not explain this higher risk of SCD in white collar workers. The potential role of other factors such as behavioural and psychosocial stressors in the workplace warrants further investigation.
